# Biomechanical Isolation is Required for Maintenance of the Cardiac Pacemaker Cell Fate

**DOI:** 10.21203/rs.3.rs-9520289/v1

**Published:** 2026-05-04

**Authors:** Michael Bressan, Ashlyn Laidman, Trevor Henley, Wei Shi, Kathryn Scherrer, Angie Mordant, Frank Conlon

**Affiliations:** University of North Carolina at Chapel Hill; University of North Carolina Chapel Hill; University of North Carolina Chapel Hill; University of Nebraska Medical Center; University of North Carolina Chapel Hill; UNC Metabolomics and Proteomics Core Facility, Department of Pharmacology, University of North Carolina at Chapel Hill, Chapel Hill, North Carolina 27599, USA.; University of North Carolina

## Abstract

Electrical impulses initiated within the sinoatrial node (SAN) drive rhythmic beating of the heart. These electrical impulses are generated by specialized cardiomyocytes termed cardiac pacemaker cells (CPCs). While the ionic mechanisms that control CPC function have long been studied, the upstream cellular events that pattern and maintain the unique electrophysiological properties of the SAN remain poorly understood. Using quantitative proteomic approaches, we have identified that developing CPCs lack fundamental components of the molecular machinery necessary to sense and respond to mechanical signaling cues. Furthermore, we have identified that ectopic activation of the core mechanotransduction pathways within CPCs induces severe SAN electrical dysfunction. Mechanistically, we demonstrate that entire systems of ion channels required for electrical oscillation and the major transcription factor networks associated with CPC cell lineage commitment are rapidly downregulated in response to cellular strain. These data reveal that the mechanical uncoupling and/or suppression of mechano-transductive signaling pathways represent a previously unrecognized critical regulatory mechanism required to support cardiac pacemaking.

## INTRODUCTION

The cardiac conduction system is responsible for initiating and coordinating the electrical activation of the heart. The most proximal component of this system is the sinoatrial node (SAN). Herein, specialized myocytes, termed cardiac pacemaker cells (CPCs), continuously oscillate between polarized and depolarized membrane potentials, resulting in the rhythmic generation of electrical impulses. Once initiated, these impulses propagate away from the SAN to activate the downstream cardiac muscle, stimulating the entire cardiac cycle.

The SAN is anatomically, functionally, and transcriptionally distinct from the atrial and ventricular working myocardium (Boyett et al., 2000; Easterling et al., 2021; James, 1977; Mandla et al., 2021; Opthof, 1988; Shiraishi et al., 1992). At the histological level, the SAN can be identified by its high content of extracellular matrix, poorly aligned muscle fibers, and significantly elevated numbers of non-myocardial interstitial cells (Boyett et al., 2000; James, 1961; Opthof, 1988). In addition, CPCs possess several hallmark features that distinguish them from the working myocardium. These include the relative paucity of contractile myofibrils, absence of t-tubules, significantly underdeveloped intercalated discs, and CPCs are far smaller than their working myocardial counterparts (Masson-Pevet et al., 1979; Masson-Pevet et al., 1984; Opthof et al., 1986). Furthermore, CPCs express unique sets of cell surface, voltage-gated, ion channels and utilize an intricate system of intracellular Ca^+2^ shuttling to spontaneously generate rhythmic electrical impulses (Alig et al., 2009; Bucchi et al., 2012; Carmeliet, 2019; DiFrancesco, 1993; Lakatta et al., 2010; Maltsev and Lakatta, 2008; Satoh, 2003).

To date, the specialized anatomical and functional characteristics present in the SAN have largely been attributed to cell fate decisions that occur during embryological development. More specifically, a set of embryologically expressed transcription factors including SHOX2, TBX3, TBX18, and ISL1 are thought to promote CPC fate acquisition and suppress maturation towards the working myocardial phenotype (Bakker et al., 2012; Blaschke et al., 2007; Christoffels et al., 2006; Espinoza-Lewis et al., 2009b; Grijalva et al., 2019; Hoogaars et al., 2007; Kapoor et al., 2012; Liang et al., 2015; Mommersteeg et al., 2010; Vedantham et al., 2015). However, the upstream molecular pathways that actively control CPC developmental cell fate decisions and/or maintain SAN function throughout life remain poorly understood.

Previously, we have shown that tissue-specific disassembly of adherens junctions that occurs during the developmental patterning of the SAN is critical for the maintenance of CPC electrical activity (Thomas et al., 2021). Additionally, we have shown that the extracellular matrix present in the embryonic SAN is far more mechanically compliant than the adjacent atrial chamber myocardium (Henley et al., 2023). Both in the context of cell-cell and cell-matrix interactions, we have shown that increasing the mechanical force applied to CPC leads to electrical dysfunction (Henley et al., 2023; Thomas et al., 2021). Collectively, these data indicated that localized differences in cardiac tissue mechanics may play a significant role in the functional patterning of the SAN. Thus, a detailed understanding of how CPCs sense and respond to the mechanical forces present within their microenvironment is necessary to create a complete mechanistic model of SAN development and homeostasis.

To address this, we set out to construct a proteomics-based profile of the intracellular signaling environment present within developing CPCs. We specifically focused on protein abundance as recent reports have noted significant divergence between differential gene expression and overall protein content in the embryonic heart (Edwards et al., 2023; Shi et al., 2021). Indeed, using mass-spectrometry, we have now identified large numbers of proteins that show differential abundances between SAN, atria, and ventricles that have not previously been described in stage-matched RNA sequencing studies (Henley et al., 2023). Among these, we found that regulatory factors specifically associated with Rho GTPase cycling are downregulated in the SAN at the protein level. Importantly, our data further demonstrate that stimulating Rho GTPase activity severely disrupts SAN electrical behavior. This occurs through a massive transcriptional response that turns off entire classes of ion channels, transcription factors, and structural genes that are required for CPC function. In agreement with these findings, we show that downstream effectors of RhoA signaling are not natively active in the developing SAN and that stimulating these factors via mechanical strain phenocopies Rho GTPase activation. Collectively, these data demonstrate that mechanically isolating CPCs is a fundamental upstream cellular event required to pattern the unique electrophysiological characteristics present in the SAN and that activation of mechanosensitive signaling modules leads to rapid and severe breakdown of cardiac pacemaking.

## RESULTS

### Proteomic analysis of the developing SAN.

**Figure 1:**
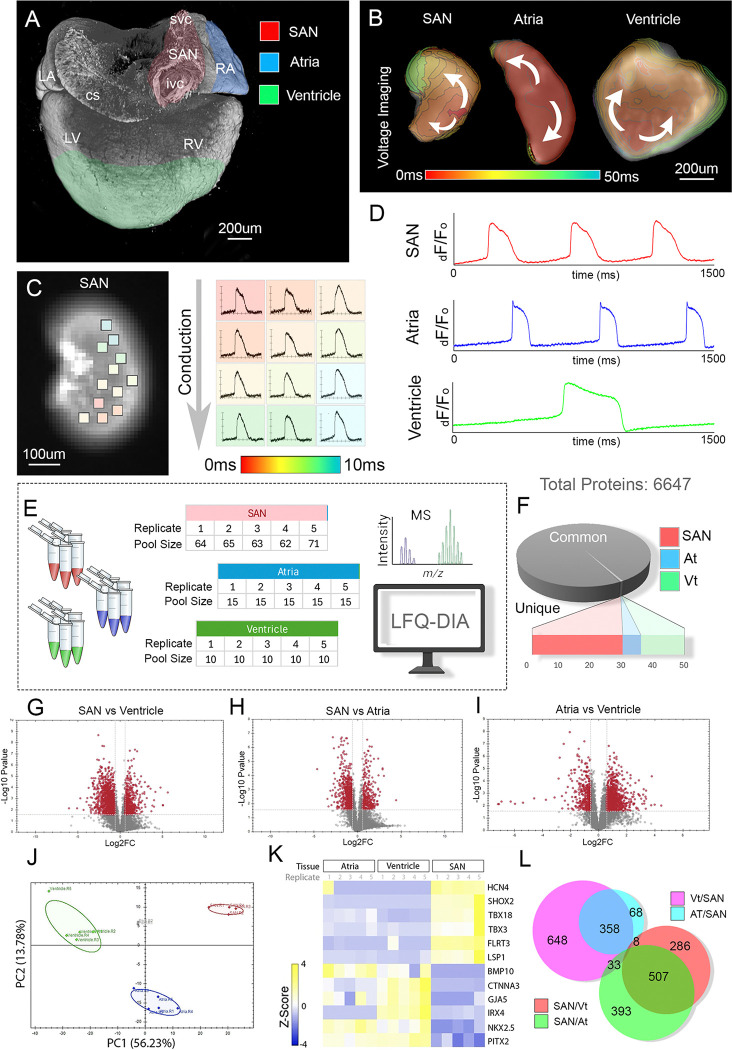
Proteomic Analysis of the Embryonic Sinoatrial Node. **A)** Dorsal view of the E6 (HH 30) embryonic heart, cardiac muscle is stained with the anti-sarcomere antibody MF20. Regions isolated for analysis, SAN (red), right atria (blue), and ventricle (green), are highlighted. **B)**Isochronal maps depicting voltage propagation across isolated SAN, atria, and ventricle (1 ms/div). **C)** Voltage Imaging of isolated SAN with action potential wave forms indicated for each boxed region. **D)** Optical recordings of action potential wave forms from SAN, atria, and ventricular samples. **E)** Schematic of sample collection and mass spectrometry. **F)**Distribution of common vs unique proteins identified. **G)** Volcano plot of differentially expressed proteins between ventricle and SAN, data points reflect Log2FC >1.0, p-values < 0.05. **H)** Volcano plot of differentially expressed proteins between atria and SAN, data points reflect Log2FC >1.0, p-values < 0.05. **I)**Volcano plot of differentially expressed proteins between atria and ventricles, data points reflect Log2FC >1.0, p-values < 0.05. **J)** Principle component analysis of sample groups: SAN (red), atria (blue), and ventricles (green). **K)** Heat map of protein expression of known SAN and working myocardial markers. **L)** Venn diagram of differentially expressed proteins between SAN, atria, and ventricle (Log2FC >1.0, p-values < 0.05). 358 proteins were upregulated in the atria and ventricle over SAN, and 507 proteins were upregulated in the SAN over the atria and ventricle. SAN – sinoatrial node, RA – right atria, LA – left atria, svc – superior vena cava, cs-coronary sinus, RV – right ventricle, LV – left ventricle.

To identify potential novel upstream regulators of CPC functional maturation, we profiled differences in protein content between the developing SAN, atria, and ventricle. To date, a quantitative proteomic analysis of the developing SAN has not been performed. This is due, in large part, to its relatively small size and poor accessibility of this tissue in most model organisms. To overcome this, we performed our analysis using the embryonic chick heart. At embryonic day 6 (E6, Hamburger Hamilton stage 30), the chick SAN displays structural and molecular characteristics comparable to mammalian systems of similar stages, and generates mature pacemaker action potentials (Bressan et al., 2013; Henley et al., 2023; Thomas et al., 2021). Importantly, the embryonic chick SAN at this stage is also relatively large (~600um × 300um) (FIG1A), containing ~8,000–10,000 cells, and can be easily isolated using microsurgical dissection. Therefore, we isolated the embryonic SAN, the free wall of the right atria, and the apex of the ventricular myocardium for analysis(FIG1A). We confirmed that each isolated tissue region displayed functional characteristics that aligned with their expected cell fate using high-speed voltage imaging. Consistent with our previous studies (Bressan et al., 2013; Henley et al., 2023; Thomas et al., 2021), voltage mapping revealed electrophysiological parameters that amtched with mature CPCs, including decreased conduction velocities and the presence of slow diastolic depolarization within isolated SAN tissue (FIG1B–D)). Furthermore, CPC-like action potential kinetics could be seen across the entire surface of SAN tissue preparations (FIG1C), suggesting little contamination from other myocardial sources.

For proteomic comparisons, we collected five biological replicates of SAN, atrial, and ventricular tissue. Each SAN biological replicate consisted of greater than 60 isolated tissue preparations, atrial replicates were generated from pools of 15 tissue preparations, and ventricular replicates were made up of 10 tissue preparations (FIG1E). To maximize proteomic depth of coverage, we employed a sequential fractionation protocol to isolate both soluble and insoluble protein fractions from each sample group. This was followed by liquid chromatography-based tandem mass spectrometry. Mass spec was performed using label-free quantification in data-independent acquisition mode (LFQ-DIA). In total, we identified 6647 proteins (Table S1), of which 99.3% were detected across all sample groups (29, 6, and 14 proteins were unique to SAN, atria, and ventricle, respectively) (FIG1F). Using significance thresholds of > 1.0 Log2 fold change, we identified 1987 proteins that were differentially expressed between ventricle and SAN, 1498 proteins that were differentially expressed between atria and SAN, and 1032 proteins that were differentially expressed between atria and ventricle (FIG1G–I, Table S1). Principal component analysis (PCA) of our sample pools revealed close segregation of 5/5 atria, 5/5 ventricle, and 4/5 SAN biological replicates (SAN outlier is shown in sFIG1B) with atria and ventricle being more closely associated along principal component 1 and SAN and ventricle more similar across principal component 2 (FIG1J). To evaluate the fidelity of our proteomic analysis, we confirmed that known positive and negative molecular markers of the SAN were differentially expressed in our data set (FIG1K). We then sought to isolate proteins that were uniquely up or downregulated in the SAN when compared to both atria and ventricle tissue. This led to the identification of 358 proteins that were more highly expressed in the atria and ventricle over the SAN, and 507 proteins that were specifically enriched in the SAN (FIG1L). In addition, we compared this proteomics dataset to our previously published transcriptomic analysis (Henley et al., 2023). This revealed that 78.9% of the proteins identified as differentially regulated between the SAN and atria were not altered at the transcript level (Log2 fold change >1.0 SAN, p-value < 0.05) (sFIG1C, Table S1). Collectively, these data identified that the SAN displays a distinct and unique proteomic profile, while also establishing a foundation for novel pathway analyses and mechanistic studies into the regulation of SAN-specific developmental and electrophysiological processes.

### Depressed small GTPase signaling in the forming SAN.

**Figure 2:**
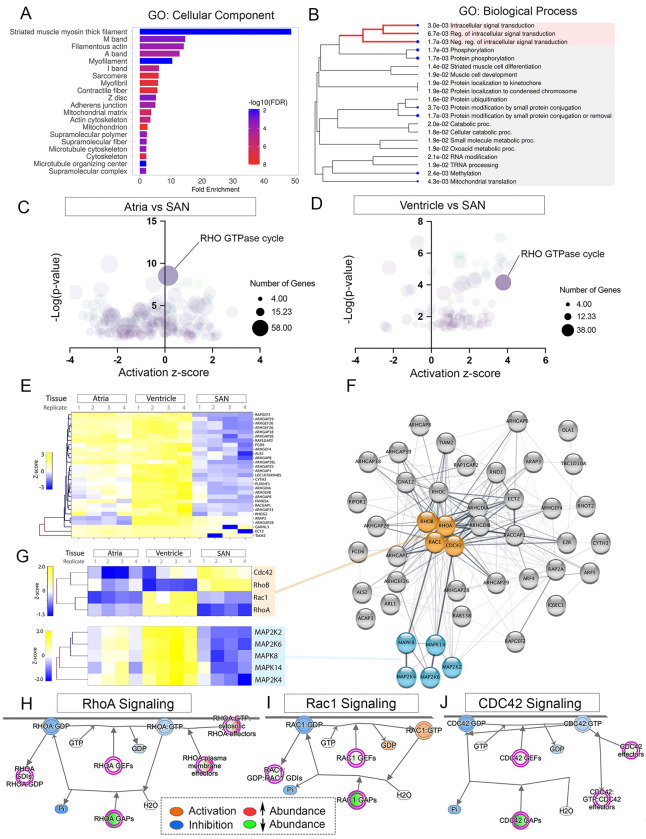
Rho GTPase cycling regulators are downregulated in the SAN. **A)** Gene Ontology (GO) term analysis (Cellular Component) of the 358 proteins enriched in the atria and ventricle over the SAN. **B)** Gene ontology (GO) term analysis (Biological Process) of the 358 proteins enriched in the atria and ventricle over the SAN. **C)** Ingenuity Pathway Analysis showing predicted pathways upstream of the differential protein expression profile observed between ventricle and SAN. **D)** Ingenuity Pathway Analysis showing predicted pathways upstream of the differential protein expression profile observed between atria and SAN. **E)** Heat map of differentially expressed RhoGAPs and RhoGEFs between atria, ventricle, and SAN. **F)** Predicted protein-protein interaction network of differentially expressed RhoGAPs and RhoGEFs. **G)** Heatmap small GTPase and MAPK effector protein abundance between atria, ventricle, and SAN. **H)** IPA-generated diagram of RhoA signaling pathway in the SAN relative to atria and ventricle. Predicated suppressed activity is indicated in blue, activation in orange. Downregulation is indicated by green. **I)** As in “H” for Rac1 signaling. **J)** As in “H” for CDC42 signaling.

We next interrogated our proteomic dataset to bioinformatically infer regulatory events that may be involved in the regional sub-specialization of cardiac function. Initially, we conducted Gene Ontology (GO) term analysis on proteins that showed differential abundance between the SAN and atrial/ventricular myocardium. GO terms associated with proteins upregulated in the SAN were enriched for Biological Processes and Cellular Components related to neuronal development, cell migration, and extracellular matrix organization (sFIG1D,E, Table S2). However, analysis of the 358 enriched in the atrial/ventricular regions revealed that factors associated with striated muscle development, adherens junction formation, and cytoskeletal organization were significantly downregulated in the SAN (FIG2A, Table S2). In addition, we noted that terms associated with intracellular signal transduction were also significantly enriched in atrial and ventricular cells when compared with the SAN (FIG2B, sFIG1F). Using our global proteomic profiles of the SAN, atrial, and ventricular myocardium we then performed Ingenuity Pathway Analysis (IPA)(Kramer et al., 2014) to generate predictions of the most significantly activated signaling pathways for each region of the heart. This revealed that RHO GTPase Cycling was predicted to be highly active in the atrial and ventricular myocardium but significantly reduced in the SAN (FIG2C,D, Table S3). Indeed, we bioinformatically isolated all of the differentially expressed proteins related to small GTPase cycling within our proteomics dataset and noted that greater than 25 GTPase Activating Proteins (GAPs) and Guanine Exchange Factors (GEFs) were downregulated in the SAN when compared with atrial and ventricular myocardium (FIG2E, sFIG1G, Table S3). We assembled these differently regulated GAP/GEFs into a predicted protein interaction network (Franceschini et al., 2013; Snel et al., 2000; Szklarczyk et al., 2025), which highlighted significant interactions with small GTPases such as RhoA, RhoB, RhoC, Rac1, or CDC42 (FIG2F,H–J). Of these factors, RhoA, RhoB, Rac1, and CDC42 were detected in our proteomics dataset, with RhoA and Rac1 downregulated in the SAN relative to atria and ventricle (FIG2G). Of note, downstream effectors of small GTPase signaling including MAP2K2, MAP2K6, MAPK8, MAPK14, and MAP2K4 were also downregulated in the SAN (FIG2G). Collectively, these data lead to the prediction that Rho, Rac1, and/or CDC42 signaling were significantly diminished in the forming SAN when compared with the atrial and ventricular chamber myocardium (FIG2H–J).

### Ectopic activation of RhoA signaling specifically disrupts SAN function.

**Figure 3:**
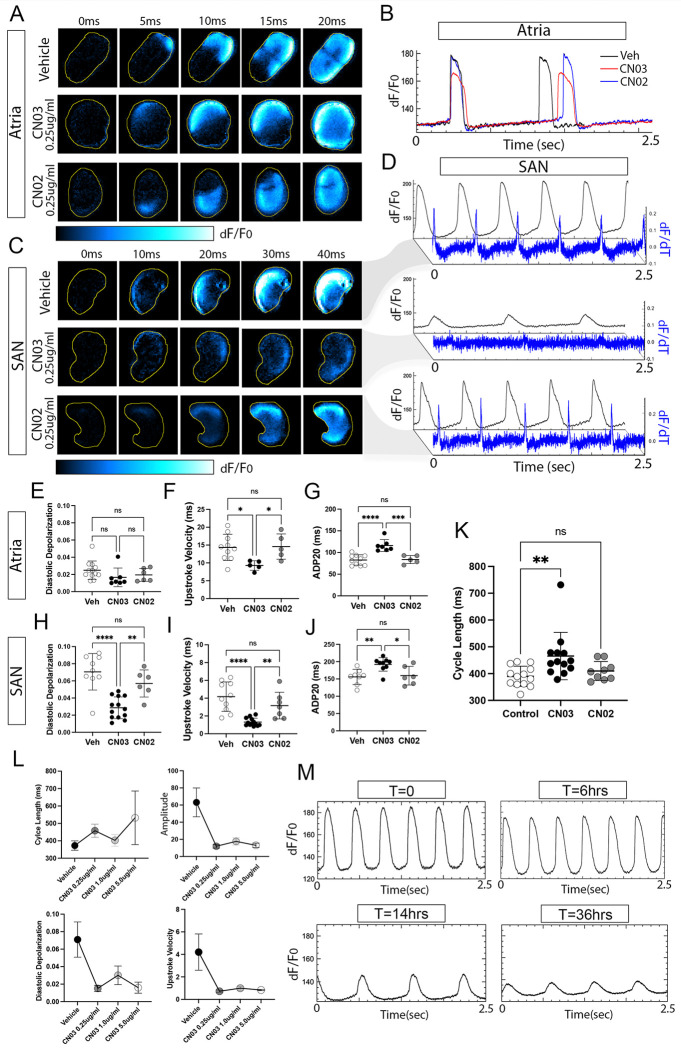
RhoA activation disrupts SAN electrical activity. **A)** Time series of voltage imaging of atrial tissue preparations following 14hrs of vehicle, 0.25ug/ml RhoA activator CN03, or 0.25ug/ml of the RAC1/CDC42 activator CN02. **B)** Optically recorded action potential traces from atrial tissue preparations treated with vehicle (black), CN03 (red), and CN02 (blue). **C)** Time series of voltage imaging of atrial tissue preparations following 14hrs of vehicle, 0.25ug/ml RhoA activator CN03, or 0.25ug/ml of the RAC1/CDC42 activator CN02. **D)** Optical recordings of action potentials (black) and dF/dT (blue) from SAN tissue preparations treated with vehicle, CN03, and CN02. **E)** Quantification of atrial diastolic depolarization in vehicle-treated (n=11), CN03-treated (n=7), or CN02-treated (n=6) samples. **F)** Quantification of atrial action potential upstroke velocity in vehicle-treated (n=11), CN03-treated (n=7), or CN02-treated (n=6) samples. **G)** Quantification of atrial action potential duration in vehicle-treated (n=11), CN03-treated (n=7), or CN02-treated (n=6) samples. **H)** Quantification of SAN diastolic depolarization in vehicle-treated (n=9), CN03-treated (n=12), or CN02-treated (n=5). **I)** Quantification of SAN sample action potential upstroke velocity in vehicle-treated (n=9), CN03-treated (n=12), or CN02-treated (n=5). **J)** Quantification of SAN sample action potential duration in vehicle-treated (n=9), CN03-treated (n=12), or CN02-treated (n=5). **K)** Quantification of SAN sample cycle length in vehicle-treated (n=12), CN03-treated (n=12), or CN02-treated (n=9). **L)** Quantification of cycle length, optical action potential amplitude, diastolic depolarization, and action potential upstroke velocity for 0.0ug/ml, 0.25ug/ml, 1.0ug/ml, and 5.0ug/ml CN03 (n=6 per condition). **M)** Optically recorded voltage changes in SAN tissue preparations cultured in 0.25ug/ml CN03 for 0, 6, 14, and 36hrs. Significance was calculated using a one-way ANOVA, p-values *<0.05, **<0.01,*** <0.001, **** < 0.0001.

Given the prediction that RhoA, Rac1, and/or CDC42 signaling may be depressed in the SAN, we tested whether ectopic activation of any of these pathways influenced electrical patterning in the heart. For these studies, we isolated SAN and atrial tissue from E6 (HH30) embryonic chick hearts and cultured them for 14hrs in vehicle, 0.25ug/ml of the RhoA-specific activator CN03(Flatau et al., 1997; Schmidt et al., 1997), or 0.25ug/ml of the Rac1/CDC42 activator CN02(Lundin et al., 2020). Following culture, we used voltage imaging to assess and quantify electrophysiological parameters. Among atrial tissue preparations, we observed no statistical differences in action potential kinetics recorded between vehicle and CN02-treated preparations (FIG3A,B,E–G, Video S1), suggesting that Rac1 and CDC42 activation did not alter atrial electrophysiology. We did observe minor, but statistically significant, decreases in action potential upstroke velocity and elongation of action potential duration in atrial preparations treated with CN03 (FIG3A,B,E–G). Similar to atrial tissue, the SAN did not display quantifiable differences in electrical activity following CN02 treatment (FIG3B,C, H–K, Video S2), again suggesting that Rac1 and CDC42 stimulation did not influence electrophysiological patterning. However, CN03 had a significant impact on SAN function (FIG3B,C, H–K, Video S2). More specifically, CN03 decreased the amplitude and dv/dt max of the SAN action potential (FIG3D), decreased the rate of slow diastolic depolarization by 2.4 fold (FIG3H), slowed upstroke velocity by 3.2 fold (FIG3I), and prolonged action potential duration by 23.2% (FIG3J). CN03 also increased the cycle length of the SAN from 390.0 +/− 36.4ms to 465.7 +/− 84.8 ms (FIG3K). These effects on the SAN electrical activity were consistent across a dose response range of CN03 concentrations from 0.25ug/ml to 5.0ug/ml (FIG3L). In addition, we performed a time series analysis and determined that CN03 did not influence SAN function over shorter time windows (less than 6 hrs), but rather the major effects of RhoA activation occurred between 6 hrs and 14 hrs of treatment and persisted for up to 36 hrs (FIG3M). Importantly, in parallel studies, we noted that suppression of RhoA signaling via treatment with 1.0ug/ml of the Rho inhibitor, CT04 (Ingallina et al., 2018), did not affect either atrial or SAN electrical activity (sFIG2, Video S3, Video S4). Thus, these data revealed that activation of RhoA, but not Rho Suppression or activation of Rac1/Cdc42, significantly disrupts SAN electrical activity. Furthermore, these data demonstrated that the SAN was far more sensitive to RhoA stimulation than the atrial myocardium, and that the ability of CN03 to influence SAN behavior occurred over 6–14 hour time window.

### RhoA signaling deactivates large portions of the SAN gene program.

**Figure 4:**
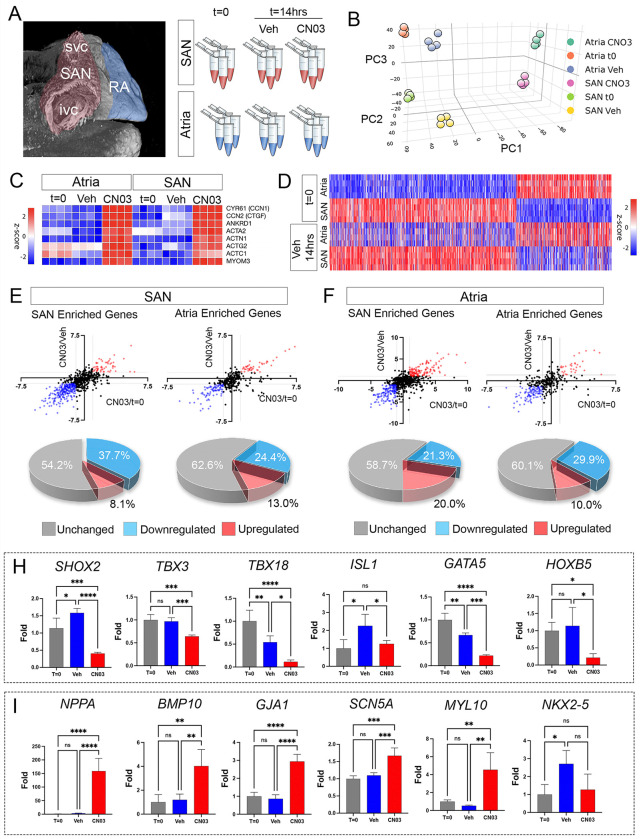
RNA sequencing analysis of SAN and atrial tissue preparations treated with the RhoA activator CN03. **A)** Dorsal view of the E6 (HH 30) embryonic heart depicting regions isolated for RNA sequencing analysis. Four biological replicates consisting of 5 pooled SAN and atrial tissue preparations were isolated for analysis. Sample groups consisted of freshly isolated tissue (t=0), cultured with vehicle for 14hrs, and cultured in 0.25ug/ml CN03 for 14hrs. **B)** Principal component analysis of RNA sequencing results. **C)** Heat map comparing gene expression of known RhoA-sensitive genes between freshly isolated, vehicle-treated, and CN03-treated atrial and SAN tissue preparations. **D)** Heatmap of differentially expressed genes between freshly isolated (t=0) atria and SAN (Log2FC >1.0, pvalue <0.05). Following 14 hrs. of vehicle treatment, overall differential gene expression profile between SAN and atria is conserved. **E)** Analysis of SAN-enriched and atrial-enriched gene (from “D”) response to CN03 treatment. Data are plotted CN03/t=0 samples (x-axis) and CN03/Veh (y-axis). Genes downregulated by CN03 vs both t=0 and Veh (Log2FC > 1.0) are indicated in blue. Genes upregulated by CN03 vs both t=0 and Veh (Log2FC > 1.0) are indicated in red. Pie charts indicate the percentage of SAN and atrial-enriched genes that were unchanged, upregulated, or downregulated following CN03 treatment when compared to both t=0 and vehicle. **F)** As in “E”, for atrial samples**. H)** Changes in SAN-enriched transcription factor expression between freshly isolated (t=0), vehicle, and CN03-treated SAN samples. **I)** Changes in selected atrial-enriched gene expression between freshly isolated (t=0), vehicle, and CN03-treated SAN samples. Statistical significance was calculated using a one-way ANOVA, p-values *<0.05, **<0.01,*** <0.001, **** < 0.0001.

Due to the 6–14 hour time window over which CN03 treatment disrupted SAN function, we hypothesized that RhoA activation was specifically impacting CPC gene expression. To test this, we collected RNA from freshly isolated SAN and atria, as well as from tissue that had either been treated for 14 hrs with vehicle or 0.25ug/ml CN03. We then performed bulk RNA sequencing to identify differentially expressed genes (FIG4A). Following sequencing, we used PCA to evaluate overall sample variance. This demonstrated that our sample pools primarily segregated based on three principal components: PC1 - CN03 treated vs untreated, PC2 - freshly isolated vs cultured, and PC3 - sample origin (SAN vs atria) (FIG4B, sFIG3A,B). To validate that CN03 was functioning as anticipated, we examined genetic targets activated by RhoA (Yu et al., 2016; Yuan et al., 2018; Zhao et al., 2014) and noted that factors including *CCN1, CTGF, ANKRD1, ACTA2, ACTN1, ACTG2, ACTC1*, and *MYOM3* were upregulated in CN03-treated atrial and SAN samples (FIG4C). To confirm that placing tissue into culture for 14 hrs. did not fundamentally alter cell fates, we compared differentially expressed genes between freshly isolated SAN and atria and vehicle-treated samples. We identified 980 differentially expressed genes (Log2FC >1.0, p-value <0.5) between freshly isolated SAN and atria (sFIG4D, Table S4). Importantly, these genes were largely unchanged between freshly isolated (t=0) and vehicle-treated (t=14hrs) SAN and atrial tissue (Pearson correlation coefficients of r=0.86 and r=0.84, respectively), suggesting that the gene programs responsible for SAN and atrial functional differences were not generally disrupted by 14 hrs of culture (FIG4D).

We next focused our analysis of the effects of RhoA activation on the genes that were differentially expressed between freshly isolated SAN and atria tissue, as these genesets likely reflect upstream regulatory processes that participate in establishing and/or maintaining the functional differences between these two tissue types. Importantly, our sequencing data revealed that 37.7% of all the genes enriched in freshly isolated SAN tissue over freshly isolated atria were downregulated by RhoA activation (FIG4E). For comparison, only 24.4% of atrial-enriched genes were down-regulated in the SAN following CN03 treatment (FIG3E). Indeed, when we specifically evaluated the top 100 SAN-enriched genes (freshly isolated SAN over freshly isolated atria) we noted that 64 of these genes were downregulated by CN03 when compared to vehicle (Log2FC > 0.6, pvalue < 0.05)(sFIG3E). Collectively, these data demonstrated that a large percentage of the SAN genetic program was negatively impacted by RhoA activation.

Given the large transcriptional changes present between vehicle and CN03-treated samples, we evaluated the transcription factor expression across sample groups (sFIG3F,G). *SHOX2, ISL1, TBX3*, and *TBX18* are among the major transcription factors associated with CPC fate acquisition (Bakker et al., 2012; Blaschke et al., 2007; Christoffels et al., 2006; Espinoza-Lewis et al., 2009b; Hoogaars et al., 2007; Hu et al., 2014; Ionta et al., 2015; Kapoor et al., 2012; Liang et al., 2015; Mommersteeg et al., 2010; Sun et al., 2007; van Eif et al., 2019; Wiese et al., 2009). Of these, *SHOX2, TBX3,* and *TBX18* were robustly downregulated by CN03 treatment (FIG4G, sFIG3F). *ISL1* showed an increase in expression following culture (vehicle-treatment over freshly isolated), however, this culture-induced increase in ISL1 expression was significantly suppressed by CN03 treatment (FIG4G, sFIG3F). We further noted that a large proportion of the SAN-enriched transcription factors identified by comparing freshly isolated SAN to atria were also downregulated by CN03 (sFIG3F). These include *Gata4*, *Gata5*, *ID1*, as well as a collection of genes within the HOXAcluste*r (HOXA2, HOXA3, HOXA4, HOXA6, HOXA7)* and the HOXB cluster (*HOXB2, HOXB3, HOXB4, HOXB5*) (FIG4G, sFIG3F). Collectively, these data indicated that core transcription factor networks associated with pacemaker cell fate commitment were rapidly downregulated by RhoA activation.

To test if CPCs were becoming more atrial-like following RhoA stimulation, we evaluated changes in genes associated with atrial fate within our dataset. Notably, major atrial-enriched genes including *NPPA, BMP10, GJA1, SCN5a*, and *MYL10* were all induced in CPCs following CN03 treatment (FIG4H). However, when we evaluated gene expression changes across our entire dataset, we observed that only 13.0% of the total number of genes enriched in the freshly isolated atria over freshly isolated SAN were induced in the SAN by CN03 treatment. Furthermore, the major cardiogenic transcription factor known to be elevated in working myocardium over CPCs, *NKX2–5 (Bressan et al., 2013; Espinoza-Lewis et al., 2009a; Hoogaars et al., 2004; van der Maarel et al., 2023; Wiese et al., 2009)*was not induced by RhoA activation (FIG4H). These data suggest that the disruption of the SAN genetic program induced by CN03 was not the result of a more general conversion of CPCs to an atrial-like phenotype, but rather RhoA stimulation influenced the expression of specific subsets of genes.

### SAN ion channel program is negatively regulated by RhoA signaling.

**Figure 5:**
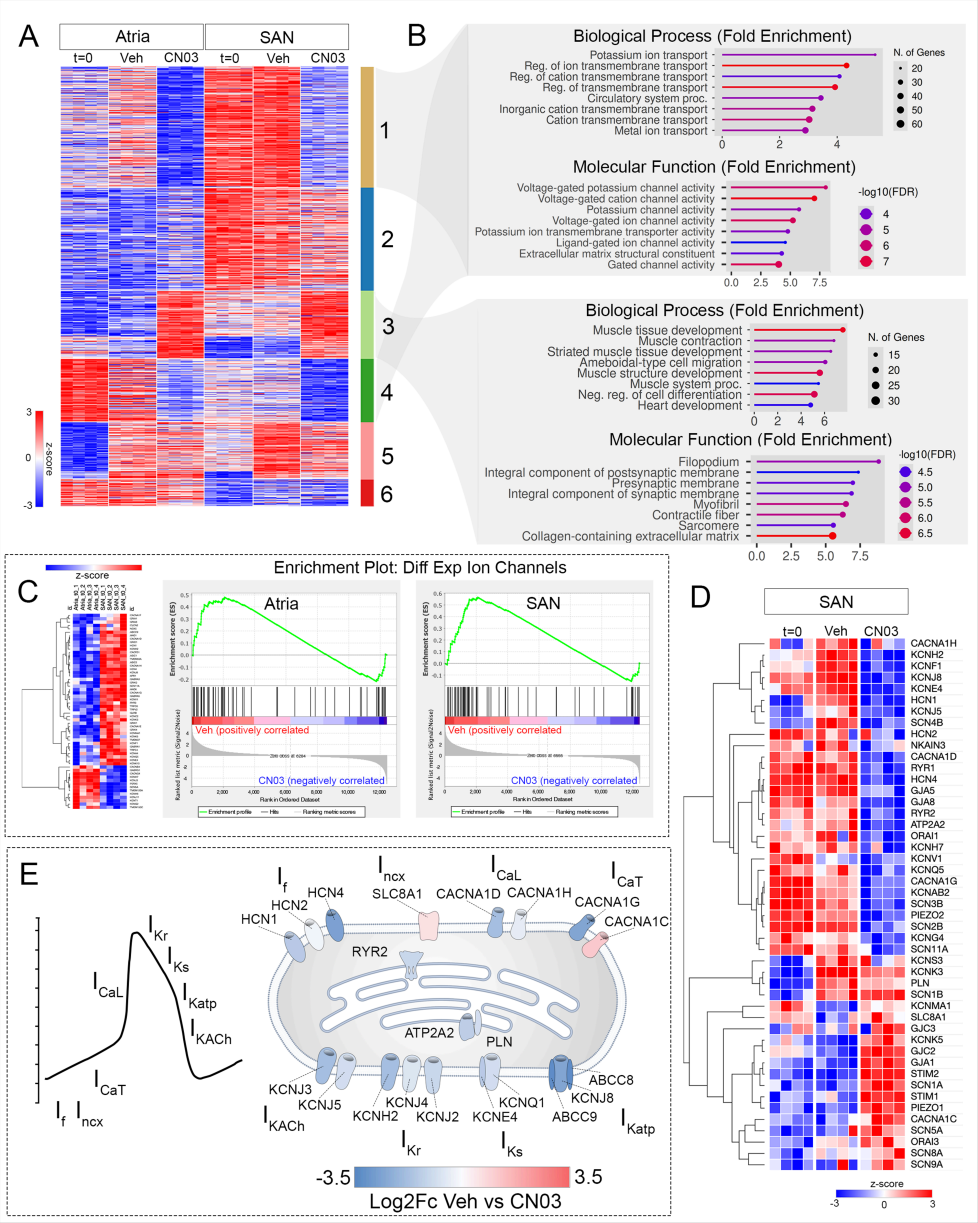
RhoA activation turns off the SAN ion channel program. **A)**K-means clustering of differentially expressed genes between freshly isolated (t=0), vehicle-treated, and CN03-treated atrial and SAN tissue samples. **B)** GO term analysis of genes downregulated by CN03 treatment (clusters 1+4 from “A”) and upregulated by CN03 treatment (cluster 3 from “A”). **C)** Gene set expression analysis (GSEA) between CN03 and vehicle treated samples. Geneset was constructed of all of the differentially expressed ion channels between freshly isolated SAN and atrial tissue. **D)** Heatmap of gene expression changes among a manually annotated list of ion channels and gap junctions between freshly isolated, vehicle, and CN03-treated SAN tissue preparations. **E)** Diagram of the ion currents that participate in the cardiac pacemaker cell action potential. Color coding denotes relative expression change (Log2FC) of the genes that encode the relevant ion channels.

To identify the specific genetic modules most heavily influenced by RhoA activation, we conducted K-means clustering of our RNA sequencing data. Herein, we identified groups of genes that demonstrated similar responses across our treatment groups. This analysis revealed 6 major gene clusters (FIG5A). Within these, clusters 1 and 4 represented genes that were downregulated in both atrial and SAN tissue following CN03 treatment. Conversely, cluster 3 represented genes that were upregulated by CN03 treatment. Using GO term analysis, we identified that genes downregulated by CN03 (clusters 1+4) were largely involved in ion transport and voltage-gated channel activity (FIG5B). In contrast, GO terms associated with striated muscle tissue development, myofibril formation, contractile fiber formation, and sarcomere formation were enriched by CN03 treatment (cluster 3)(FIG5C). Based on the abundance of GO terms associated with voltage-gated ion channel activity and the severe changes in action potential kinetics we noted among SANs treated with CN03, we bioinformatically isolated all of the differentially expressed ion channels between freshly isolated SAN and atria and packaged them into a custom geneset (FIG5D, Table S4). We then performed Gene Set Enrichment Analysis (GSEA)(Subramanian et al., 2005) on sequencing data collected from atria and SAN tissue treated with vehicle or CN03. Overwhelmingly, ion channels within this gene set were positively correlated with vehicle treatment and negatively correlated with CN03 treatment in both atrial and SAN tissue (FIG5E). These data demonstrate that the composition of the ion channels expressed by these tissues is highly sensitive to RhoA signaling. Furthermore, this sensitivity is universal, occurring in both SAN and atrial tissue (FIG5C, sFIG4A,B), suggesting that the influence RhoA has on ion channel expression is not dependent on tissue lineage.

We next expanded our expression analysis to include a manually annotated list of ion channels, pumps, and gap junctional proteins (FIG5C, Table S4). These genes were not exclusively chosen for analysis based on differential expression between atria and SAN, but were selected because they encode channels known to participate in pacemaker vs atrial electrical activity and/or mechanically-activated ion currents (FIG5D). Among these channels, we observed a significant number of ion currents associated with the pacemaker action potential that were downregulated. Specifically, *HCN1, HCN2, HCN4,* which carry the pacemaker funny current, *CACNA1D, CACNA1H,* which carry the L-type Calcium current, *CACNA1G,* which carries T-type calcium current, *RYR2, ATP2A2,* which are required for the CPC calcium clock cycling, and *KCNJ3, KCNJ5, KCNH2, KCNJ4, KCNJ2, KCNQ1*, and *KCNJ8* which are involved in repolarization were all coordinately downregulated by RhoA activation with in SAN (FIG5D,E) (cardiac pacemaker currents are reviewed in (Aziz et al., 2018; DiFrancesco, 1993; Lakatta et al., 2010; Maltsev and Lakatta, 2012; Tsutsui et al., 2018)). As noted above, many of the channels were also downregulated in atrial tissue following CN03 treatment (sFIG4C,D). In addition, several factors associated with atrial electrical activity including the sodium channel *SCN5A* and gap junction GJA1 were induced in the SAN by RhoA stimulation, suggesting that functional markers that delineate SAN from atria are dynamically regulated by RhoA signaling. To validate these findings, we conducted RNAscope-based fluorescent *in situ* hybridization on SAN tissue preparations that had been treated either with vehicle or 0.25ug/mL CN03 for 14 hrs. In agreement with our RNA sequencing data, RNAscope confirmed that *HCN4* transcript levels were decreased and GJA1 transcript levels were increased by CN03 treatment (sFIG4E,F). Collectively, these data demonstrate that RhoA is a potent negative regulator of CPC fate, deactivating entire classes of genes that functionally discriminate the SAN from the working myocardium.

### RhoA Signaling activates classical mechanosensitive transcriptional regulators in the myocardium.

**Figure 6:**
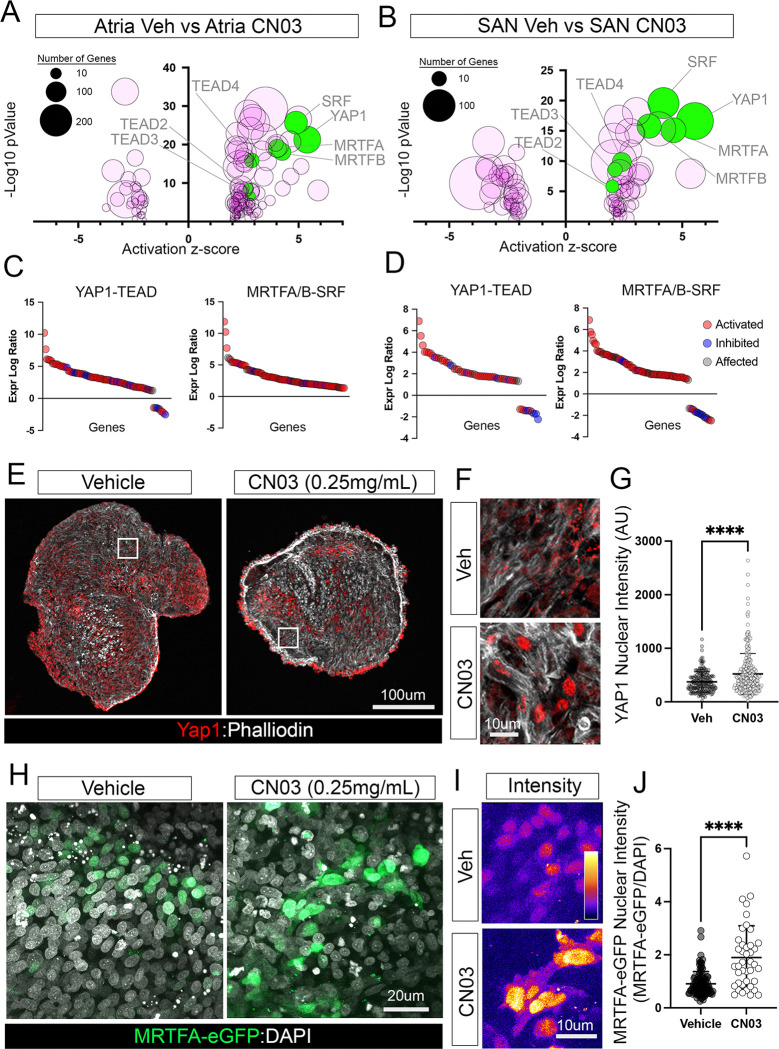
RhoA activates classical mechanosensitive transcriptional regulators in the embryonic myocardium. **A)** Ranking of predicted transcription factor activation (Activation score, p-value, and number of downstream genes that changed expression) in vehicle vs. CN03-treated atrial tissue. **B)** Ranking of predicted transcription factor activation (Activation score, p-value, and number of downstream genes that changed expression) in vehicle vs CN03-treated SAN tissue. **C)** Log2FC expression ratio (CN03/Veh) of genes predicted to be activated (red), inhibited (blue), or affected (grey) by the YAP1-TEAD and MRTFA/B-SRF transcription factors in atrial samples. **D)** Log2FC expression ratio (CN03/Veh) of genes predicted to be activated (red), inhibited (blue), or affected (grey) by the YAP1-TEAD and MRTFA/B-SRF transcription factors in SAN samples. **E)** Immunohistochemistry for YAP1 (red) and phalloidin (white) in Vehicle and CN03-treated SAN samples. **F)** Higher magnification images of regions from “E” demonstrating increased nuclear YAP1 staining in CN03-treated samples. **G)** Quantification of YAP1 Nuclear intensity between Vehicle and CN03-treated SAN tissue. **H)** MRTFA-eGFP expression in vehicle and CN03-treated myocardium. Cells are counterstained with DAPI (white). **I)** Higher magnification intensity plots of MRTFA localization. **J)** Quantification of MRTFA-eGFP nuclear intensity. Statistical significance was calculated using a Welch’s t-test, p-values **** < 0.0001.

RhoA is a master regulator of mechnotransduction in a wide variety of cell types (Lessey et al., 2012; Ohashi et al., 2017). RhoA also acts downstream of G protein-coupled receptor activity and oxidative stress in the heart(Miyamoto, 2024). We therefore evaluated our RNA sequencing data to predict the potential pathways highly activated in CPCs following RhoA stimulation. We used Ingenuity Pathway Analysis to generate an unbiased ranking of altered transcription factor activity based on the all the differentially expressed genes affected by CN03 treatment. Herein, we noted a clear molecular profile consistent with activated mechnotransduction. More specifically, YAP1, MRTFA, MRTFB, and SRF were each among the top ten transcription factors predicted to be activated by CN03 in the myocardium (FIG6A,B, Table S5). YAP1 is a well-described mechanostransduction-sensitive transcription factor that moves to the nucleus during periods of increased cellular strain where it interacts with TEAD transcriptional co-regulators to alter gene expression(Dupont et al., 2011; Halder et al., 2012; Panciera et al., 2017; Totaro et al., 2018). Of note, TEAD2, TEAD3, and TEAD4 were also predicted to be activated based on our sequencing data sets (FIG6A,B, Table S5). Similarly, MRTFs respond to actin dynamics. High levels of F-actin during cytoskeletal elaboration and stiffening allow for MRTFA/B nuclear trafficking, where they co-regulate SRF(McGee et al., 2011; Miralles et al., 2003; Montel et al., 2019). SRF, in turn, controls the expression of the myofibril and cytoskeletal gene program (Cenik et al., 2016; Deshpande et al., 2022; Guo et al., 2018; Yuxuan Guo 2019).

To validate these bioinformatic predictions, we treated SAN tissue preparations with vehicle or CN03 for 14 hrs. and examined YAP1 and MRTFA localization. Following treatment with CN03, immunohistochemical staining for YAP1 demonstrated significant increase in nuclear intensity compared to vehicle controls (FIG6E–G). Consistent with these findings, we also observed a significant increase in MRTFA nuclear localization following CN03 treatment. Due to a lack of a validated antibody for MRTFA/B in the chick, we evaluated activation of this pathway using an MRTFA-eGFP fusion protein. We overexpressed MRTFA-eGFP in the developing heart using *in vivo* transfection (Goudy et al., 2019) and then isolated SAN tissue preparations and treated them with CN03 for 14 hrs. Quantification of MRTFA-eGFP intensity revealed a 2.1 fold increase in nuclear localization in CN03-treated samples when compared to vehicle (FIG6H–J). Collectively, these data confirmed that pharmacological activation of RhoA signaling in the myocardium was sufficient to induce nuclear accumulation of transcription factors associated with mechanically stimulated changes in gene expression.

### Suppression of cellular strain response is required to maintain the CPC phenotype.

**Figure 7:**
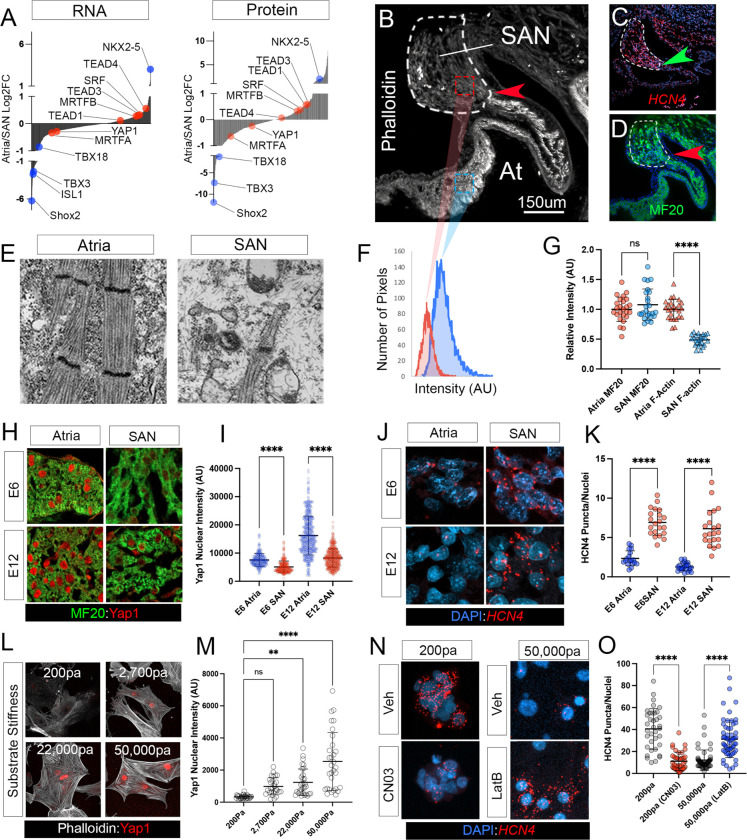
Mechanical stress phenocopies RhoA activation in the SAN. **A)** Transcript and protein expression (Log2 fold atria/SAN) of all transcription factors detected in RNA sequencing and mass spectrometry studies. Known SAN and atrial enriched factors are highlighted in blue. Mechanosensative factors associated with YAP/TEAD and MRTF/SRF are highlighted in red. **B)** Phalloidin staining of the SAN/atrial junction in an E6 (HH30) embryonic heart. SAN is indicated by dashed region. Red arrow demarcates the boundary of phalloidin staining at the SAN/atrial junction. **C)** RNAscope for *HCN4* transcript. Green arrowhead demarcates the SAN/atrial boundary noted in “B”. **D)**Immunohistochemistry using the anti-sarcomere antibody MF20. Red arrow demarcates the SAN/atrial boundary noted in “B”. **E)** Transmission electron micrographs of atrial and SAN myocytes demonstrating significantly higher F-actin associated with the forming sarcomeres in the atria. **F)** Phalloidin intensity profiles from the SAN (red) and atrial (blue) regions indicated in “B”. **G)** Quantification of MF20 and phalloidin intensity within the atria and SAN (n=25 regions of interest (ROI), imaged across 6 biological replicates). **H)** Immunohistochemistry of YAP1 in the E6 (HH30) and E12 (HH38) SAN and atria. **I)** Quantification of YAP1 nuclear intensity in the E6 SAN (n=326) vs atria (n=234) and E12 SAN (n=493) vs atria (n= 489). Data were collected from 6 biological replicates. **J)** RNAscope-based fluorescent *in situ* hybridization for *HCN4* in the E6 and E12 SAN vs atria. **K)** Quantification of HCN4 RNAscope in the E6 SAN (n=21 ROIs) vs atria (n= 17 ROIs) and E12 SAN (n=21 ) vs atria (n=24 ROIs). **L)** Immunohistochemistry for YAP1 and Phalloidin in CPCs isolated from the E6 heart and plated on polyacrylamide hydrogels of ~200, 2,700, 22,000, and 50,000Pa. for 48 hrs**M)** Quantification of nuclear YAP1 intensity in CPCs plated on 200Pa (n=29), 2,700Pa (n=28), 22,000Pa (n=31) and 50,000Pa (n=31) hydrogels. **N)** RNAscope-based fluorescent *in situ* hybridization for *HCN4* in E6 CPCs plated on 200 or 50,000Pa hydrogels with or without CN03 and Latrunculin B treatment. **O)** Quantification *HCN4* puncta in CPCs plated on 200Pa hydrogels treated with vehicle (n=36) vs 0.25ug/ml CN03 (n=40) and CPCs plated on 50,000Pa hydrogels treated with vehicle (n=54) vs 1.0uM Latrunculin B (n=58). Statistical significance was calculated using Welch’s t-tests, p-values **<0.01, **** < 0.0001.

The above findings strongly implicate RhoA signaling as a negative regulator of CPC functional patterning. These findings suggest that RhoA-mediated mechano-signaling should be natively inactive for normal SAN development to occur. To test this, we initially evaluated the expression of SRF, MRTFA, MRTFB, YAP1, TEAD1, TEAD3, and TEAD4 in our RNA sequencing and proteomic data sets to determine whether any of these factors were downregulated in the SAN vs the working myocardium. However, we noted no differential expression, either at the transcript or protein level, of any of these factors between SAN and atria (using Log2 fold change cut-offs of 0.6 and p-values of 0.05) (FIG7A). Therefore, we next examined activation of these factors. The ratio of F-actin to G-actin determines MRTFA/B localization and co-regulates SRF function(Miralles et al., 2003). Importantly, when we examined F-actin levels via phalloidin staining, we noted that F-actin was markedly lower in the SAN compared to the atria(FIG7B). Indeed, a clearly defined border of F-actin intensity was detected at the SAN/atrial junction. This border exactly corresponded with the transition point at which the SAN functional marker *HCN4* went from low expression in the atrial myocardium to high expression in the SAN (FIG7C,D). We further confirmed the lower levels of F-actin content in the SAN using transmission electron microscopy (FIG7E) and by quantifying phalloidin staining intensity across 6 biological replicates (FIG7F,G). This revealed that F-actin was significantly reduced in the SAN.

We next examined the localization of YAP1 in the SAN at two developmental stages. At E6 (HH30), when the embryonic SAN is fully functional but undergoing significant cellular remodeling (Henley et al., 2023), YAP1 nuclear intensity was significantly lower in the *HCN4*-positive SAN when compared to the adjacent atrial myocardium (FIG7H–K). Consistent with these findings, at E12 (HH38), following the completion of SAN morphogenesis (Henley et al., 2023), YAP1 nuclear accumulation remained significantly higher in the atria than the SAN (FIG7H–K). These data demonstrate that the *HCN4*+ CPC myocardium has far lower F-actin content and YAP1 nuclear localization than the atria.

Importantly, the above data are highly consistent with our previous findings demonstrating that the embryonic SAN is far more mechanically compliant than the working myocardium (Henley et al., 2023; Thomas et al., 2021)and collectively suggest that cellular strain is an important determinant of SAN patterning. Therefore, we tested whether increasing mechanical stress was sufficient to phenocopy RhoA pharmacological activation. For these studies, we isolated and cultured primary embryonic CPCs on polyacrylamide hydrogels of increasing stiffness. When CPCs were cultured on hydrogels of ~200Pa, equivalent to the passive stiffness of the E6 SAN (Henley et al., 2023), we noted extremely low levels of both F-actin and nuclear YAP1. However, increasing hydrogel stiffness to ~2,700Pa, equivalent to the passive stiffness of the E6 atria (Henley et al., 2023), or above, resulted a dose-dependent increase in YAP1 nuclear localization and extensive F-actin elaboration (FIG7L,M). We next tested how cellular strain impacted gene regulation by scoring *HCN4* expression across a range of substrate stiffnesses. Here, we plated primary embryonic CPCs on hydrogels of 200Pa or 50,000Pa and monitored *HCN4* expression using RNAscope. This analysis demonstrated that *HCN4* transcript was 70.0% lower on stiff 50,000Pa hydrogels compared to compliant 200Pa hydrogels (FIG7N,O). Of note, treating CPCs on soft 200Pa hydrogels with 0.25ug/ml CN03 decreased *HCN4* expression to levels consistent with those observed on 50,000Pa hydrogels (FIG7N,O). In addition, we treated CPCs on 50,000Pa hydrogels with the actin de-polymerization antagonist, Latrunculin B. Importantly, on 50,000Pa hydrogels, Latrunculin B fully rescued *HCN4* expression to levels consistent with soft hydrogels (FIG7N,O). Collectively, these data demonstrate that the forming SAN displays a molecular signature consistent with low mechanical stimulation. Furthermore, these data indicate that increasing substrate stiffness is sufficient to disrupt the CPC molecular phenotype and that this disruption can be rescued by pharmacologically targeting pathways activated by cellular strain.

## DISCUSSION

The ionic mechanisms that control cardiac pacemaking have long been of interest, yet the upstream cellular events that control the acquisition and maintenance of the CPC lineage have remained poorly understood. Here, we have identified that biomechanical isolation of CPCs serves as a major regulatory event that controls large portions of the SAN gene program. Indeed, our findings demonstrate that the activation of a single mechanosensitive signaling cascade leads to rapid and severe dysregulation of SAN electrical impulse initiation. Of note, our current findings are highly consistent with our previous studies that demonstrate CPCs uniquely disassemble their force-generating cell-cell junctions during cardiac development (Thomas et al., 2021) and that the SAN requires the formation of a relatively soft local extracellular matrix (Henley et al., 2023). Expanding on these previous findings, our current study demonstrates that suppression of cellular strain responses is required to sustain expression of large numbers of transcriptional regulators, ion channels, and structural genes needed for SAN activity. These findings have significant ramifications, indicating that cellular mechanics are a principal determinant of SAN patterning and emphasize that mechanical stimulation should be a primary design consideration as future bioengineering techniques advance towards the creation of cellular-based pacemakers for *in vivo* clinical use (Cingolani, 2015; Cingolani et al., 2018; Komosa et al., 2021; Miake et al., 2002; Vedantham, 2015).

Previous work has suggested that a collection of transcriptional regulators including ISL1, SHOX2, TBX3, and TBX18, function to suppress the maturation of CPCs, leaving these cells in a more juvenile or less well-developed cardiomyocyte state (Christoffels et al., 2010; Liang et al., 2017). Our current study adds to this model by demonstrating that maintenance of these transcription factors, as well as a host of other SAN-enriched transcription regulators, requires low levels of RhoA activation. These findings further indicate that expression of the SAN transcriptional network is fairly plastic and can be dynamically modulated by local mechanical stimuli.

Of particular note, hallmark features of the CPC phenotype, and a primary reason why these cells are often referred to as juvenile, is that these cells contain few sarcomeres and generally lack mature intercalated discs(Miyamoto et al., 2002; Opthof et al., 1987; Shimada et al., 2004). Our proteomic evaluation of the developing SAN largely captured these features, indicating that proteins related to striated muscle myosin thick filament, M band, filamentous actin, A band, I band, sarcomere, Z disc, and adherens junction formation were all significantly downregulated in the SAN when compared with the atrial and ventricular myocardium (FIG2A). Importantly, our proteomic analysis also established that multiple factors associated with Rho GTPase cycling were significantly downregulated during formation of SAN, and our RNA sequencing demonstrated that ectopic activation of RhoA within CPCs leads to the upregulation of genes associated with GO terms including muscle contraction, myofibril formation, contractile fiber formation, and sarcomere formation (FIG5B). Collectively, these data indicate that the establishment of cellular features that have historically been used to define the CPC lineage are directly regulated by Rho signaling tone. Herein, we would also note that the vast majority of differentially expressed Rho regulatory factors identified in this study were not altered at the transcript level. This is the likely reason that previous RNA sequencing approaches have not identified the Rho signaling pathway as critical for SAN formation - highlighting the utility of a proteomics-based approach.

Given current literature, the link between RhoA activation and cytoskeletal gene upregulation is not overly surprising (Arai et al., 2002; Burridge and Chrzanowska-Wodnicka, 1996; Mack et al., 2001; Sotiropoulos et al., 1999). However, our data demonstrate a strong negative impact on SAN physiological function following stimulation with CN03. Consistent with this physiological disruption, CN03 treatment resulted in the broad downregulation of ion channels required for CPC electrical oscillation. These data suggest that the core ion channel program that encodes the capacity for rhythmic electrical oscillation in the heart is mechanosensitive. Thus, our data highlight an integrative mechanism of ion channel regulation in the heart, whereby factors responsible for initiating electrical impulses would be inactivated in mechanically strained areas of the working myocardium, and specifically confined to regions of low force generation such as the SAN. Therefore, under normal conditions, basic tissue mechanics would help suppress the activation of ectopic and potentially arrhythmogenic electrical impulses in the working myocardium by locally suppressing the expression of the genes responsible for pacemaking.

Additionally, our data would predict that alterations to SAN tissue mechanics over the life span of an organism should lead to CPC failure. Here, we would emphasize that SAN dysfunction is extremely prevalent in humans, affecting ~1:600 adults over the age of 60 (Jensen et al., 2014). While our current mechanistic understanding of SAN pathophysiology is limited, fibrotic remodeling, which would be predicted to stiffen the SAN, has long been associated with pacemaker-related disease (Csepe et al., 2015; Ho and Sánchez-Quintana, 2016; Shiraishi et al., 1992). Using a developmental system, our data have now uncovered a large number of mechanosensitive regulatory factors that may represent novel candidate pathways for SAN-disease modeling. Indeed, our data strongly agree with previous work that demonstrates overexpression of RhoA across the entire adult mouse heart results in specific defects in heart rate regulation (Sah et al., 1999), and that constitutively activated YAP1 in the cardiac conduction system caused pacemaker-related arrhythmias (Zheng et al., 2022). Adding to these previous reports, our RhoA stimulation studies demonstrate that much of the SAN genetic program is not fixed via cell fate decisions but responds and adapts dynamically to mechanosensitive signaling pathways. Thus, our data provide a mechanistic framework linking the developmental mechanisms that establish CPC functional maturation to conditions that should cause SAN failure in adults. Importantly, our data also demonstrate that SAN-enriched genes, such as *HCN4*, can be reactivated, even under conditions of high mechanical load, by chemically disrupting intracellular actin-dynamics. These findings suggest that pharmacological approaches targeting specific components of the cell strain response machinery may serve to prevent or reverse SAN dysfunction, leading to novel potential treatment strategies for human arrhythmogenesis.

## MATERIALS and METHODS

### Experimental Model and Study Participants Details

Fertilized chicken eggs were obtained from Allen Harim Hatchery. Upon arrival, eggs were placed at 16C until ready for use. Eggs were then placed in a humidified incubator (Hova-Bator, Genesis, 1588) until the desired developmental Hamburger-Hamiliton Stage. All procedures were approved by the University of North Carolina’s American Association for Accreditation of Laboratory of Animal Care Committee.

### Method Details

#### Liquid Chromatography-Based Tandem Mass Spectrometry

##### Protein Isolation

SAN, atrial, and ventricular tissues were collected, snap-frozen in liquid nitrogen, and stored at −80 °C until use. A sequential extraction protocol was employed to fractionate soluble and insoluble proteins with modifications(Barallobre-Barreiro et al., 2013; Barallobre-Barreiro et al., 2021). To ensure comparable extraction efficiency, atrial and ventricular tissues were finely minced with sterile surgical scissors and homogenized using a disposable pestle until fragments were reduced to a size comparable to SAN.

Homogenized atrial, ventricular, and SAN tissues were immersed in ice-cold PBS supplemented with protease and phosphatase inhibitors and 25 mM EDTA, rotated at 4 °C for 5 min, and centrifuged at 15,000 × g for 1 min at 4 °C. The supernatant was discarded. This wash step was repeated four times to remove plasma contaminants. Following the final wash, tissues were resuspended in 0.5 mL NaCl extraction buffer (0.5 M NaCl, 10 mM Tris-HCl, pH 7.5, protease and phosphatase inhibitors, and 25 mM EDTA), transferred to a new tube, and gently rotated (~25 rpm) for 3 h at room temperature. Samples were then centrifuged at 18,000 × g for 10 min at 4 °C, and the supernatant (SN-1) was collected and stored at −80 °C.

The remaining pellets were briefly washed with 1 mL NaCl extraction buffer and 1 mL ddH_2_O, and the supernatants were discarded. 0.5 mL high-salt buffer (50 mM Tris-HCl, 3 M NaCl, 25 mM EDTA, 0.25% (w/v) CHAPS, pH 7.5, protease and phosphatase inhibitors) was added, and samples were rotated at 4 °C for 5 min, followed by centrifugation at 18,000 × g for 20 min at 4 °C. The supernatant (SN-2) was collected and stored at −80 °C. Tissue pellets were then washed briefly with ddH_2_O, and 0.5 mL freshly prepared guanidine extraction buffer (6 M guanidine-HCl, pH 9.0) was added. Samples were vortexed with a TOMY shaker for 1 h at room temperature and centrifuged at 18,000 × g for 20 min at 4 °C. The supernatant (SN-3) was collected and stored at −80 °C. Fractions SN-1, SN-2, and SN-3 were combined and designated as the soluble fraction.

The remaining insoluble pellets were washed sequentially with 1 mL 1% Triton X-100 buffer (supplemented with protease and phosphatase inhibitors and 25 mM EDTA) for 30 min at room temperature and 1 mL ddH_2_O for 10 min, repeated three times. Samples were centrifuged at 4 °C for 5 min after each wash, and supernatants were discarded. Following the final wash, pellets were resuspended in 0.25 mL guanidine extraction buffer (4 M guanidine-HCl, 50 mM sodium acetate, 25 mM EDTA, protease and phosphatase inhibitors, pH 5.8) and shaken vigorously at room temperature for 72 h using a TOMY shaker. Samples were then heated at 95 °C for 5 min and sonicated for 15 seconds using a Microplate Horn System with chiller (QSONICA). The heat and sonication cycle was repeated three times. Samples were centrifuged at 18,000 × g for 15 min at 4 °C, and the supernatant was collected as the insoluble fraction.

Both soluble and insoluble fractions were precipitated using the methanol/chloroform method (Wessel and Flugge, 1984). Soluble protein pellets were stored at −80 °C until mass spectrometry processing. Insoluble protein pellets were resuspended in 40 μL resuspension buffer (2 M urea in 50 mM ammonium bicarbonate) and deglycosylated using a Protein Deglycosylation Kit (NEB, P6044) per the manufacturer’s instructions, followed by methanol/chloroform precipitation. Final protein pellets were stored at −80 °C until mass spectrometry analysis.

##### LC-MS/MS sample preparation and data acquisition

Protein pellets were resuspended in 2M Urea, 50 mM ammonium bicarbonate. Resuspended samples were reduced with 5mM DTT and alkylated with 15mM iodoacetamide for 45 min at room temperature. Samples were subjected to digestion with LysC (Wako) at 37°C for 2h and trypsin (Promega) overnight at 37°C at a 1:50 enzyme:protein ratio. The resulting peptides were acidified to 0.5% trifluoroacetic acid and desalted using Thermo desalting spin columns. Eluates were dried via vacuum centrifugation and peptide concentration was determined via Pierce Quantitative Fluorometric Assay. All samples were normalized to 0.09 μg/μl. Samples (14.5 ul) were spiked with 0.5 ul iRT peptide (Biognosys). For quality control, a pooled sample was created by combining 2 ul from each sample.

Samples were analyzed in a randomized order by LC-MS/MS using an Ultimate 3000 coupled to an Exploris 480 mass spectrometer (Thermo Scientific). The pooled sample was analyzed at the beginning and end of the sequence. Samples were injected onto an IonOpticks Aurora series 2 C18 column (75 μm id × 15 cm, 1.6 μm particle size) and separated over a 120 min method. The gradient for separation consisted of 3–41% mobile phase B at a 250 nl/min flow rate, where mobile phase A was 0.1% formic acid in water and mobile phase B consisted of 0.1% formic acid in 80% ACN. The Exploris 480 was operated in product ion scan mode for Data Independent Acquisition (DIA).

Resolution for the precursor scan (m/z 375–1500) was set to 120,000, with AGC set to 300%. Following the full MS scan, a product ion scan was collected with a resolution set to 15,000, and normalized AGC set to 200%. The normalized collision energy was set to 30% for HCD. Peptide match was set to preferred, and precursors with unknown charge or a charge state of 1 and ≥ 7 were excluded.

##### Proteomics Data Analysis

Raw data files were processed using Spectronaut (v18; Biognosys) and searched against the Uniprot Gallus gallus database (UP000000539, containing 51,565 entries, downloaded January 2024) and the MaxQuant common contaminants database (245 entries). The following settings were used: enzyme specificity set to trypsin, up to two missed cleavages allowed, cysteine carbamidomethylation set as a fixed modification, methionine oxidation and N-terminal acetylation set as variable modifications. A false discovery rate (FDR) of 1% was used to filter all data, and single hit proteins were removed. iRT calibration was enabled and median normalization was applied based on proteins identified across all samples. Imputation was disabled. Un-paired student’s t-tests were conducted and p-values, q-values (adjusted p-values, BH-correction), and log2 fold change ratios were calculated in Spectronaut. Proteins with an absolute log2 fold change ≥ 1 and a q-value < 0.05 were considered significant. Targeted pathway enrichment analysis was run using Ingenuity Pathway Analysis software (Kramer et al., 2014) (QIAGEN Inc., https://digitalinsights.qiagen.com/IPA).

#### RNA Sequencing

##### RNA Isolation

SAN and atrial tissue were isolated from HH 30 chick embryos using microdissection as shown in FIG4A. Each sample pool consisted of 5 SAN and 5 atrial tissue preparations. We generated 4 biological replicates for six experimental groups: freshly isolated SAN and atrial tissue, SAN and atria cultured with 1ul molecular biology water (vehicle) in complete primary culture media (15% FBS (Avantor Seradigm, 97068–085), 1% penicillin-streptomycin (Gibco, 15140122), and 1% amphotericin B (Gibco, 15290026) in DMEM/F12 (Gibco, 11330032)) for 14 hours, and SAN and atria cultured with 0.25ug/mL of CN03 (Cytoskeleton Inc, CN03) in complete media for 14 hours. For RNA isolation, pooled tissue was transferred to 500 uL of sterile Hanks’ Balanced Salt Solution (HBSS) (Gibco, 14175–095). Tissue was then spun down for 10 minutes at 8,000 rcf, the top 150uL of HBSS was removed, samples were flash frozen in a solution of dry ice and ethanol, and stored at −80C.

Samples were thawed on ice and HBSS was removed, leaving the sample pellet. Then 500uL of TRIzol (Invitrogen, 15596026) was added and the sample was spun through a homogenizer column (Invitrogen, 1283–026) for 2 minutes at 12000×g at room temperature (RT) twice to ensure complete homogenization. An additional 500uL of TRIzol was added and the sample vortexed for 5 seconds. Next, 200uL of ice-cold chloroform (Sigma, 25666) was added to the sample, the Eppendorf was shaken vigorously for 15 seconds, and then the solution incubated at RT for 3 minutes. The sample was centrifuged at 12000×g for 15 minutes at 4C. The top aqueous layer (~400–600uL) was collected, 500uL of 2-proponal was added, and the sample gently inverted 2x. Then, 0.5 ug/uL of glycogen (Thermo, R0561) was added and the sample gently inverted 2x. Samples incubated at −20C for 24 hours.

Next, RNA was precipitated by centrifuging the sample at 12000×g for 15 minutes at 4C. The supernatant was discarded and 1 mL of 75% ethanol was added to wash the pellet. The sample was then centrifuged at 12000×g for 5 minutes at 4C and the supernatant was removed. The pellet air dried for 7 minutes and then resuspended in 25 uL of DEPC water (GrowCells, UPW).

Library preparation was conducted using the Illumina Stranded Total RNA-SEQ with Ribo-Zero Kit and sequencing was performed on an Illumina NovaSeq X Plus system (pair end × 150bp). RNA sequencing quality assurance was run using FastQC (http://www.bioinformatics.babraham.ac.uk/projects/fastqc). Index construction and read mapping were performed using Kallisto (Bray et al., 2016). RNA sequencing data filtering was conducted using edgeR (Chen et al., 2025), and differential gene expression analysis was completed using limma. Upstream pathway prediction was performed using Ingenuity Pathway Analysis software(Kramer et al., 2014) (QIAGEN Inc., https://digitalinsights.qiagen.com/IPA).

#### Tissue and Cell Culture

##### Tissue Preparations

SAN and right atrial (RA) explants were manually dissected from E6 embryonic chicks in sterile warmed HBSS^++^ (Gibco, 14025–076). Explants were placed in 35mm glass bottom dishes (Cellvis, D35–14-1.5-N) for culture at 37C in 2mL complete media for the times indicated in figures.

##### Primary Cardiac Pacemaker Cell Culture

SAN explants were manually dissected from E6 embryonic chicks in sterile warmed HBSS^−−^ (Gibco, 14175103). Explants were then transferred to pre-warmed dissociation solution composed of 17% Trypsin-EDTA (Gibco, 25200056) in HBSS^−−^ and supplemented with 8.5 ug/mL of collagenase/dispase (MilliporeSigma, 1026963800). The solution was agitated every 5 minutes until explants were no longer visible, for a total of ~10 minutes. The dissociation solution was then neutralized with twice the volume of pre-warmed complete media and centrifuged for 10 minutes at 1,000 rcf to pellet the cells. The cell pellet was resuspended in complete media and cell count determined via hemocytometer. To deplete non-myocyte cells from the suspension, cells were pre-plated on fibronectin-coated glass plates for 45 minutes to allow for non-myocardial cell adhesion, and then the supernatant was transferred to fibronectin-coated polyacrylamide hydrogels.

##### Inhibitor and Activator Treatments

The RhoA specific activator CN03 (Cytoskeleton Inc., CN03) was reconstituted to a concentration of 0.1 ug/uL in sterile water and incubated on ice for 10 minutes. The stock solution was then diluted to a working concentration of 0.25 ug/mL in complete media and used for cell culture.

The Rac1/CDC42 activator CN02 (Cytoskeleton Inc., CN02) was reconstituted to a concentration of 0.1ug/ul. The stock solution was then diluted to a working concentration of 0.25ug/mL in complete media and used for cell culture.

The RhoA specific inhibitor CT04 (Cytoskeleton Inc., CT04) was reconstituted to a concentration of 0.1 ug/uL in sterile water. The stock solution was then diluted to a working concentration of 1.0 ug/mL in complete media and used for cell culture.

Latrunculin B (Sigma, 428020) was reconstituted to a concentration of 1.0 mm in DMSO. The stock solution was then diluted to a working concentration of 1.0uM in complete media and used for cell culture.

#### Polyacrylamide Hydrogels

To prepare the 35 mm tissue culture dishes (Matsunami Glass, D35–14-0-U), the dishes were plasma activated for 2 minutes (Harrick Plasma, PDC-001), and coated with 0.5% APTES (Sigma, A3648) for 30 minutes, excess APTES was rinsed off with molecular biology grade water, and the APTES was fixed with 0.5% glutaraldehyde for 30 minutes. The glutaraldehyde was rinsed off, and the culture dishes were dried completely. Next, the hydrogel solutions were prepared according to the table below, poured into the culture dishes, covered with a quartz coverslip (Chemglass Life Sciences, CGQ06001) coated in SigmaCoat (Millipore, SL2), and allowed to rest for 10 minutes for the hydrogel to solidify. Reagents for the hydrogels were as follows: 40% acrylamide (Millipore, A4058), 2% bisacrylamide (Millipore, M1533), 10x PBS (Gibco, 14200–075), TEMED (Sigma, T9281), 1% APS (Millipore, A3678). To add a surface adhesion molecule to the hydrogel for cell culture, the hydrogel surface was activated in a UVO Machine (Jelight, Model 24) for 200 seconds and then a solution of 10 mg/mL EDC (Millipore, 03450) and 17.5 mg/mL NHS (SigmaAldrich, 130672) in molecular biology water was immediately added, the quartz coverslip removed, and the solution rocked for 15 minutes. Excess EDC/NHS was rinsed off with molecular biology water. Finally, hydrogels were incubated with 3 ug/cm^2^ fibronectin (Millipore, F2006) overnight in a cell culture incubator (BenchMark, SureTherm45 H3565–45). The fibronectin was rinsed off, hydrogels were covered with 1x PBS, UV sterilized for 15 minutes, and stored at 4C for up to 2 weeks before use.

**Table:** 

Stiffness (Pa)	40% Acrylamide (uL)	2% Bisacrylamide (uL)	10x PBS (uL)	Molecular Biology Water (uL)	TEMED (uL)	1% APS (uL)
**200**	150	60	200	1388	2	200
**2,700**	375	35	200	1188	2	200
**22,000**	375	250	200	973	2	200
**50,000**	500	500	200	598	2	200

#### Cryosections

After culture in complete media (see above), SAN and atria tissue preparations were fixed in 2% PFA (ThermoFisher, J19943-K2) for 30 minutes at room temperature and then the PFA was washed off via 3×5 min washes in PBS. Tissue preperations were moved through a sucrose gradient of 10%, 20%, 30% and 50% sucrose (ThermoScientific, 036508.A1) - 1 hour per solution. Samples were then embedded in OCT (Tissue-Tek, 4583), flash frozen using dry ice and 100% ethanol, and stored at −80C. The explants were then sectioned using a Leica Cryostat CM1850 into 12 um sections on glass coverslips (Fisher, 22–037-246). Coverslips were stored at −80C until ready for use.

#### RNAscope

##### Cryosection and Paraffin Slides Pretreatment

Detection of RNA was conducted using RNAscope Multiplex Fluorescent V2 Assay kit (ACD, 323270). Pretreatment steps varied by embedding method. If samples were from cryosectioned slides, they were removed from −80C and thawed for 10 minutes at room temperature then rinsed in PBS for 5 minutes. If the samples were from paraffin sections, they were de-paraffined by incubating for 90 minutes at 60C, then moved to a solution of 100% Xylene for two 5-minute washes and rinsed in 100% ethanol for two 5-minute washes.

Slides were incubated with 3% hydrogen peroxide (322335) for 10 minutes then rinsed off with DEPC water (GrowCells, UPW). For target retrieval, slides were incubated in 1x target retrieval solution (322000) at 110C for 15 minutes, then dipped into room temperature DEPC water several times. Next, slides were incubated in 100% ethanol for 3 minutes at room temperature and allowed to air dry for 10 minutes. A hydrophobic marker was used to outline the tissue samples and allowed to dry. Protease Plus (32233) was added for 30 minutes at 40C to perforate cell membranes. Slides were dipped into DEPC water several times to rinse off the protease.

##### CPCs on Polyacrylamide Gels Pretreatment

Detection of RNA was conducted using RNAscope Multiplex Fluorescent V2 Assay kit (ACD, 323270). Hydrogel were first dehydrated in an ethanol gradient in PBS of 15%, 25%, 50%, 70%, 85%, 100% for 10 minutes at each step at 4C, then left overnight at −20C in 100% ethanol. Samples were then rehydrated in the exact reverse ethanol gradient to 100% PBS. Then hydrogen peroxide (322335) was added for 10 minutes at room temperature and washed off with DEPC water. Next, protease III (322337) was added for 10 minutes in a 1:15 dilution in DEPC-PBS and washed off with DEPC-PBS.

##### Hybridization and Amplification

The probes, either HCN4 (569141-C3) or GJA1 (807611-C3) were added in a 1:50 ration with probe diluent (300041) and allowed to incubate for 2 hours at 40C in a humid chamber. The samples were rinsed off in 1x wash buffer (310091) and placed in 5x SSC buffer (Invitrogen, 15557–036) overnight.

For the next steps, all rinse steps consisted of 2×2 minutes of incubation in 1x wash buffer. Samples were rinsed before incubating in Amp1 (323101) for 30 minutes at 40C. Then the samples were rinsed and Amp2 (323102) was added for 30 minutes at 40C. The samples were rinsed again and Amp3 (323103) was added for 15 minutes at 40C. The samples were rinsed and the appropriate developer (323106) was added for 15 minutes at 40C. The samples were rinsed again and the specified fluorophore, either Cy5 (75–000203) or Cy3 (TS-000202), was added in a 1:1000 dilution in TSA buffer (322809) and incubated for 30 minutes at 40C. Samples were rinsed and HRP blocker (323107) was added for 15 minutes at 40C. The samples were rinsed and a 1:1000 dilution of DAPI (ThermoFisher, 62248) in PBS was added for 5 minutes at room temperature. DAPI was then washed off with PBS. If on slides, coverslips (Fisher, 12–545-C) were mounted using anti-fade fluorescence mounting media (Abcam, AB104135) and sealed with clear nail polish. Samples were stored at 4C until imaging.

#### Immunohistochemistry

For tissue preparations, samples were blocked in for 1 hour in 1% BSA (SigmaAldrich, 1003356189) and 0.1% Tween-20 (ThermoScientific, 85113) in PBS. Then, primary antibodies were added overnight at 4C and rinsed off with PBS. Secondary antibodies and DAPI were added overnight at 4C and rinsed off with PBS.

For CPCs on polyacrylamide hydrogels, samples were blocked for 1 hour in 1% BSA and 0.1% Tween-20 in PBS. Then, primary antibodies were added overnight at 4C and rinsed off with PBS. Secondary antibodies were added for 1 hour at room temperature and washed off with three 5 min washes in PBS.

The following primary antibodies were used: 1:200 YAP1 (Cell Signal, D8H1X), 1:500 MF20 (Invitrogen, 14–6503-80). The counter stains were used: 1:500 Phalloidin-647 (Invitrogen, A22287), 1:1000 DAPI (ThermoFisher, 62248). Alexa Flour 488 (Invitrogen, A21141) and Alexa Flour 568 (Invitrogen, A21124) secondary antibodies were used at a concentration of 1:500.

#### Voltage Imaging

To conduct voltage imaging of SAN, atria, and ventricle, samples were bathed in a staining solution containing 10 uM Di-4-ANEPPS (Invitrogen, D1199) and 2 uM blebbistatin (Sigma, B0560) in warm HBSS^++^ (Gibco, 14025–076). Samples were incubated in the staining solution for 10 minutes at 37C. Explants were then transferred to a custom-built imaging chamber containing HBSS^++^ maintained at 35C +/− 1C using a Warner TC-344C temperature controller. Samples were illuminated with a Lex2 led light source (SciMedia) and imaging was then conducted at 2000 fps using an MiCam05 camera system (SciMedia). Action potential traces were then analyzed using BrainVision software (SciMedia) and the ImageJ plugin Spiky(Pasqualin et al., 2022).

#### *In vivo* Transfections

The method for *in vivo* embryonic chicken transfections has been previously described(Goudy et al., 2019). Briefly, expression plasmids were packaged by making a solution of 8% lipofectamine 3000 (Invitrogen, 100022050) in 37C opti-MEM (Gibco, 31985–070) and a solution of 2 ug/25 uL of the appropriate DNA plasmid in 37C opti-MEM. The two solutions were allowed to equilibrate at room temperature for 5 minutes before being combined. After combining the lipofectamine and the plasmid, the solution was incubated at room temperature for an additional 5 minutes. A 1:10 dilution of Fastgreen to trace solution during microinjections. The packaged plasmid was then backloaded into a pulled glass capillary (World Precision Instruments, TW100F-4) made using micropipette needle puller (World Precision Instruments, PUL1000). Then ~2 uL of transfection reagent was microinjected into the pericardial space of windowed E3 embryonic chicken eggs using a pressure microinjector (Eppendorf, FentoJet4). Following the injection, ~1 mL of HBSS^++^ (Gibco, 14025–076) was added to the embryo to prevent drying, tape was placed over the window, and embryos were returned to a humidified 37C incubator (Hova-Bator, Genesis, 1588) until desired stage.

The MRTFA-eGFP construct was built by fusing eGFP to the C-terminus of chick MRTFA using gene synthesis.

#### Confocal Microscopy

All immunofluorescence and RNAscope images were collected using either a Zeiss LSM 800 or a Zeiss LSM 980 super-resolution confocal imaging system. Image quantification was conducted using ImageJ v2.16.0/1.54p or Imaris 3/4D image visualization and analysis software (Oxford Instruments).

#### Transmission Electron Microscopy

Samples were prepared for TEM as described previously(Thomas et al., 2021). Briefly, E6 embryos were fixed overnight in 4% PFA at 4C. Embryos were then embedded in 3% low melting temperature agarose and cut into 200um sections using a vibratome. Sections were then post-fixed in 4% PFA, 1% glutaraldehyde, and 0.1 M sodium phosphate for 3 days at 4C. Following this post-fixation, sections were stained with 1% osmium tetroxide, 1.25% potassium ferrocyanide, 0.1 M sodium phosphate buffer and dehydrated through an ethanol series. Sections were then embedded in PolyBed 812 epoxy resin (08792–1; Polysciences) and cut to a thickness of 70–80 nm. Sections were mounted on copper grids and further stained with 4% aqueous uranyl acetate for 12 min followed by lead citrate for 8 min. Imaging was performed using a Tecnai 12 G2 CCD camera (Model 794) mounted on a JOEL JEM-1230 transmission electron microscope.

#### Quantification and Statistical Analysis

Sample numbers and statistical significance for each assay are indicated in the text and/or figure legends. Software used for proteomic and RNA sequencing analysis are described in the corresponding methods sections above. For non-proteomic and non-transcriptomic evaluation, statistical analysis was performed using Prism 10 (graphpad). As indicated in the corresponding figure legends, statistical comparisons were performed using either a two tailed Pearsons Correlation Coefficient, a one-way ANOVA, or a Welch’s t-test. Reported p-values are shown as *<0.05, **<0.01,*** <0.001, **** < 0.0001. All individual data points are plotted along with the mean (bars) and standard deviation (brackets).

## Supplementary Material

Supplementary Files

This is a list of supplementary files associated with this preprint. Click to download.

• TableS2.xlsx

• TableS4.xlsx

• TableS3.xlsx

• TableS1.xlsx

• VideoS1.mov

• VideoS3.mov

• VideoS2.mov

• VideoS4.mov

• TableS5.xlsx

• LaidmanetalSuppMaterials.docx

## Data Availability

Raw proteomic data has been deposited in the PRoteomics IDEntification Database (PRIDE) under accession number PXD075315. RNA-seq raw data has been deposited in the Gene Expression Omnibus (GEO) database under accession number GSE320003. No original code was written for this manuscript.
